# Characterization of rhizosphere bacterial communities in oilseed rape cultivars with different susceptibility to *Plasmodiophora brassicae* infection

**DOI:** 10.3389/fpls.2024.1496770

**Published:** 2025-01-06

**Authors:** Yue Deng, Wenxian Wu, Xiaoqing Huang, Xiaoxiang Yang, Yaoyin Yu, Zhongmei Zhang, Zijin Hu, Xiquan Zhou, Kang Zhou, Yong Liu, Lei Zhang

**Affiliations:** ^1^ Institute of Plant Protection, Sichuan Academy of Agricultural Sciences, Chengdu, China; ^2^ Key Laboratory of Integrated Pest Management on Crops in Southwest, Ministry of Agriculture and Rural Affairs, Chengdu, China; ^3^ Anhui Province Key Laboratory of Environmental Hormone and Reproduction, Fuyang Normal University, Fuyang, China; ^4^ Anhui Province Key Laboratory of Embryo Development and Reproductive Regulation, Fuyang Normal University, Fuyang, China

**Keywords:** rhizosphere microbiome, *Plasmodiophora brassicae*, susceptible cultivar, resistant cultivar, microbial metabolism, nitrogen cycle

## Abstract

Rhizosphere microbiomes are constantly mobilized during plant–pathogen interactions, and this, in turn, affects their interactions. However, few studies have examined the activities of rhizosphere microbiomes in plants with different susceptibilities to soil-borne pathogens, especially those that cause clubroot disease. In this study, we compared the rhizosphere bacterial community in response to infection of *Plasmodiophora brassicae* among the four different clubroot susceptibility cultivars of oilseed rape (*Brassica napus*). Our results revealed obvious differences in the responses of rhizosphere bacterial community to the *P. brassicae* infection between the four cultivars of oilseed rape. Several bacterial genera that are associated with the nitrogen cycle, including *Limnobacter*, *Thiobacillus*, *Anaeromyxobacter*, *Nitrosomonas*, *Tumebacillus*, and *Halomonas*, showed significantly different changes between susceptible and resistant cultivars in the presence of *P. brassicae* infection. Moreover, increased connectedness and robustness were exhibited in the rhizosphere bacterial community co-occurrence network in clubroot-susceptible cultivars that were infected with *P. brassicae*, while only slight changes were observed in clubroot-resistant cultivars. Metagenomic analysis of microbial metabolism also indicated differences in the rhizosphere bacterial community between susceptible and resistant cultivars that were infected with *P. brassicae*. Functional analysis of the nitrogen cycle showed that genes related to nitrification (*nxrB*) were upregulated in susceptible cultivars, while genes related to assimilatory nitrate reduction (*nasA*, *narB*, and *nirA*) were upregulated in resistant cultivars that were infected with *P. brassicae*. These findings indicate that the synthesis and assimilation process of NO_3_^-^ content were promoted in susceptible and resistant cultivars, respectively. Our study revealed differences in the characteristics of rhizosphere bacterial communities in response to *P. brassicae* infection between clubroot-susceptible and clubroot-resistant cultivars as well as the potential impact of these differences on the plant–*P. brassicae* interaction.

## 1 Introduction

Plant rhizosphere microbiomes are increasingly being studied in recent years in relation to their contributions to various aspects of plant growth, development, and health (Berendsen et al., 2012; Bardgett and van der Putten, 2014; Saleem et al., 2019). Rhizosphere microbiomes are sensitive to alterations in biotic and abiotic factors, including plant development, infection by pathogens, and disorders in soil properties, resulting in a highly dynamic and diverse microbial community throughout the entire life cycle of plants (Zegeye et al., 2019; Panke-Buisse et al., 2015; Kwak et al., 2018; Sun et al., 2020). Moreover, rhizosphere microbiomes are responses to plant/soil-borne pathogen interactions and have been widely studied in recent years. Furthermore, there is a two-way effect between microbial community assemblage and plant/pathogen interaction. In the plant/pathogen systems, plants secrete root exudates and recruit specific microbial communities that confer them with disease resistance according to the “cry for help” theory (Hu et al., 2020, 2018). Similarly, some microbes are conducive to pathogen invasion through nutritional complementarity feedback mechanisms (Li et al., 2019; Pacheco et al., 2019; Kramer et al., 2020). Changes in the microbial community of diseased plants compared with those of healthy plants are external manifestations of disease processes, and this contributes to variations in microbial metabolisms associated with energy and material metabolic cycles, such as carbon and nitrogen cycles (Cao et al., 2024). Therefore, recent studies have focused mainly on identifying certain microbial taxa in rhizosphere microbiomes that contribute to crop health by affecting microbial metabolism or inhibiting pathogen growth. These microorganisms are considered potential tools for soil-borne disease control and sustainable farming and have been widely studied in multiple crops (Sun et al., 2022; Zhang et al., 2022; Compant et al., 2019). However, relatively limited studies have focused on the impact of changes in rhizosphere microbiomes in response to plant/pathogen interactions on the development of crop disease.

Clubroot, caused by the obligate protist, *Plasmodiophora brassicae* (*P. brassicae*), is a soil-borne disease that threatens the production of Cruciferous crops worldwide, as it results in a significant reduction of 40% to 60% in both crop yield and quality (Javed et al., 2023; Chai et al., 2014). The typical symptoms of clubroot disease are the presence of root galls as well as wilting and stunting of the above-ground parts of the plant (Schuller and Ludwig-Müller, 2016; Kageyama and Asano, 2009). Traditional measures for clubroot control cannot achieve the goals of eradication due to the long survival times (5–20 years) of resting spores in the soil (Donald and Porter, 2014). Moreover, severely affected fields are unsuitable for crop cultivation for extended periods of time (Dixon, 2009). Genetic resistance is presently being considered the most economical and effective approach for clubroot control worldwide (Diederichsen et al., 2009; Rahman et al., 2014). Multiple potential clubroot resistance genes (CR gene) in *Brassica* crops that are involved in modulating disease resistance responses to *P. brassicae* infections have been identified using next-generation sequencing (NGS) and other “omics”-based methods (Nagaoka et al., 2010; Hasan et al., 2021). Furthermore, numerous clubroot-resistant cultivars of *Brassica* crops have been bred and promoted commercially, including Huashuang 5R and Huayouza 62R (Zhan et al., 2015; Shah et al., 2019). *Brassica* crops with CR loci have been shown to successfully resist *P. brassicae* infection through regulating the plant innate immunity (Zhou et al., 2020). Differences in clubroot resistance between susceptible and resistant cultivars were directly reflected in plant roots, including transcription, proteins, and metabolism (Chen et al., 2015; Zhang et al., 2016; Pedras et al., 2008; Cao et al., 2008; Li et al., 2022). However, studies on whether these CR genes participate in interactions between host root and soil microbiome during the clubroot disease process are limited.

Recent studies on clubroot have indicated a distinct shift in microbial communities of *Brassica* crops when infected with *P. brassicae* (Kang et al., 2024; Wu et al., 2020). Moreover, variations in microbial community diversity have been shown to be correlated with clubroot disease severity and are highly sensitive as indicated by the microbial community in response to infections with *P. brassicae* (Lebreton et al., 2019; Liu et al., 2024). Changes in the microbial community due to host/*P. brassicae* interactions also differ in multiple situations, including disease resistance, pathotype, soil property, and fertilization (Lebreton et al., 2019; Liu et al., 2024; Cordero-Elvia et al., 2024; Gazengel et al., 2021). A more diverse microbial community also appeared to have a more obvious effect in promoting clubroot occurrence; however, this effect varied between susceptible and resistant cultivars (Wang et al., 2023; Daval et al., 2020). Moreover, the positive impact of nitrogen supply on clubroot occurrence also varies between susceptible and resistant cultivars (Gazengel et al., 2021). These phenomena suggest that clubroot resistance mechanisms in resistant cultivar may participate in the interaction within plants, pathogens, and soil microbiomes and may play an important role in shaping microbial communities. Although several studies have shown significant differences in root performance between clubroot-susceptible and clubroot-resistant cultivars when infected with *P. brassicae*, it is still unclear how clubroot-resistant cultivars manipulate the shaping of microbial communities based on their resistance mechanisms when infected with *P. brassicae*.

Additionally, we selected four cultivars of *Brassica napus* with different clubroot resistance levels to investigate whether clubroot resistance mechanisms affect the response of the rhizosphere microbiomes to plant/*P. brassicae* interactions. Based on 16S rRNA and metagenomic sequencing, we revealed the differences in microbial community, interaction within microbial communities, and microbial metabolisms of rhizosphere microbiomes between clubroot-susceptible and clubroot-resistant cultivars with or without *P. brassicae* infection. Our results suggest that those obvious differences in the rhizosphere microbiomes between two type cultivars may be caused by a resistant mechanism based on CR genes, which further affects the plant/*P. brassicae* interactions. Our study will help broaden the strategies for clubroot resistance breeding of oilseed rape and lay the foundation for utilizing soil microbial communities to control the occurrence of clubroot disease.

## 2 Materials and methods

### 2.1 Biological materials and pathogen inoculation

The cultivars of oilseed rape (*Brassica napus* subs. napus, hybrid) C36, H62, H62R, and Menh, as well as *P. brassicae* pathotype 4 isolate (Williams, 1966) were used in this study (**Table 1**). C36, also referred to as Chuanyou 36, was provided by the Crop Research Institute, Sichuan Academy of Agricultural Sciences (China) (Jiang et al., 2011). H62 and H62R, referred to as Huayouza62 (clubroot susceptible) and Huayouza62R (clubroot resistant), were provided by Prof. Chunyu Zhang of the College of Plant Science and Technology, Huazhong Agricultural University (China) (Li et al., 2021). Menh, referred to as Menhir (clubroot resistant), was provided by Norddeutsche Pflanzenzucht Hans Georg Lembke KG (NPZ) (Germany, https://www.proplanta.de/pflanzenbauberater/sorten/menhir-winterraps-hauptfruchtanbau_sks_4351raw1.html). Meanwhile, cultivars C36 and H62 are conventional hybrids without any clubroot resistance genes. H62R was generated by crossbreeding with H62 (*Brassica napus*, recipient parent) and CR Shinki (Chinese cabbage, *CRb*, donor parent). Menh was generated by crossbreeding with clubroot-resistant (*P. brassicae* pathotype 3) Mendel. Resting spores were extracted from galled root tissue collected from *Brassica napus* ‘Chuanyou 81’ in Shifang City, Sichuan Province. Resting spore suspensions were prepared as described in a previous study (Strelkov et al., 2006). The spore suspension was adjusted to a final concentration of 10^7^ spores/mL. Each plant was inoculated with 10 mL of resting spore suspension. The resting spore suspension was injected into the soil close to the plant using a 10-mL syringe to ensure successful infection.

**Table 1:** Summary of oilseed rape cultivars used in this study.

Sample	Year	Resource	CR locus	Gene source	Reference
Chuanyou36 (C36)	2011	China	No	–	Jiang et al., 2011
Huayouza62 (H62)	2011	China	No	–	–
Huayouza62R (H62R)	2021	China	*CRb*	Gelria R	Li et al., 2021
Menhir	2015	Germany	Unknown	Unknown	North German plant breeding Hans-Georg Lembke KG

### 2.2 Experimental design and rhizosphere soil sample collection

All experiments were conducted in a greenhouse (23°C, 16-h light/8-h dark). Organic matter soil was purchased from a local market and directly used for plant cultivation without sterilization. Four cultivars of oilseed rape were also planted separately in organic matter soil in plugs (size: 540 mm × 280 mm), with each plug containing 21 holes for sowing oilseed rape. Each cultivar of oilseed rape was planted with a total of six plugs. Three plugs of plants per cultivar with a total of 63 plants were inoculated by resting spore suspensions of *P. brassicae* at 7 days; the rest of the three plugs of plants were treated with sterile water as the control treatment. For the *P. brassicae*-treated group, clubroot incidence (CI) and the disease severity index (DSI) of clubroot in each plug were calculated after 40 days of inoculation, and the rhizosphere soil of all diseased plants in the same plug were collected simultaneously into a single bag (Kuginuki et al., 1999). Regarding the control treatment, the rhizosphere soil of all plants in the same plug was also collected simultaneously into a single bag. Finally, a total of 24 rhizosphere soil samples were collected, and each treatment contained three replicates for the subsequent sequencing.

Collection and pretreatment of rhizosphere soil were done as described in a previous study (Edwards et al., 2015). The soil that remained tightly adhered to the roots after intense shaking was used as the rhizosphere soil sample. Root samples were collected into a 50-mL centrifuge tube with 25 mL of 1× PBS solution (137 mM NaCl, 2.7 mM KCl, 10 mM Na_2_HPO_4_·12H_2_O, and 2 mM KH_2_PO_4_). The mixture was sonicated at 40 Hz for 1 min and then shaken to separate rhizosphere soil from the roots. The rhizosphere soil was then transferred to a new sterile 50-mL centrifuge tube and centrifuged at 9,000 rpm for 5 min, after which the precipitated rhizosphere soil was subjected to freeze drying (BILON-FD80AD, Shanghai Bilang Instrument Manufacturing Co., Ltd., China). The dry rhizosphere soil was then homogenized by grinding (Tissuelyser-48, Jingxin, China). Finally, the processed samples were stored at -80°C for the subsequent 16S rRNA and metagenome sequencing.

### 2.3 DNA extraction and 16S rRNA sequencing

Microbial DNA was extracted from 5 g of rhizosphere soil samples of oilseed rape using the E.Z.N.A.^®^ stool DNA Kit (Omega Bio-tek, Norcross, GA, U.S.) according to the manufacturer’s instructions. DNA samples were prepared and stored at -80°C for the subsequent 16S rRNA and metagenomic sequencing. The V3–V4 region of the 16S rRNA gene was PCR-amplified (95°C for 2 min, followed by 25 cycles at 95°C for 30 s, 55°C for 30 s, 72°C for 30 s, and a final extension at 72°C for 5 min) to investigate bacterial communities using the primers 338F (5′-ACTCCTACGGGAGGCAGCA-3′) and 806R (5′-GGACTACHVGGGTWTCTAAT-3′) (Mori et al., 2013). PCR reactions were performed in triplicate 20-μL mixtures containing 4 μL of 5× FastPfu buffer, 2 μL of 2.5 mM dNTPs, 0.8 μL of each primer (5 μM), 0.4 μL of FastPfu Polymerase, and 10 ng of template DNA. The PCR products were detected using 1.5% agarose gel electrophoresis and further purified using an AxyPrepTM DNA Gel Extraction Kit (Axygen Scientific, USA). PCR products were quantified using Qubit^®^3.0 (Life Invitrogen) and pooled in equimolar concentrations of 10 ng/μL. Paired-end sequencing was performed on an Illumina HiSeq 2500 platform at Beijing Biomarker Technologies Co., Ltd., Beijing, China. Microbial bioinformatic analysis was performed using QIIME 2 2021.11 (Bolyen et al., 2019). The raw sequencing data was demultiplexed and filtered using the q2-demux plugin followed by denoising with DADA2 (Callahan et al., 2016). The phylogenetic affiliation of each 16S rRNA gene sequence was analyzed using RDP Classifier (http://rdp.cme.msu.edu/) against the silva (SSU132) 16S rRNA database using a confidence threshold of 70% (Amato et al., 2013).

### 2.4 Metagenomic sequencing

Total DNA was also extracted from the above-mentioned rhizosphere soil samples using the E.Z.N.A.^®^ Viral DNA Kit (Omega Bio-tek, Norcross, GA, USA) according to the manufacturer’s protocols. High-quality DNA sample (OD260/280 = 1.8–2.2, OD260/230 ≥ 2.0) was used to construct a sequencing library. Metagenomic shotgun sequencing libraries were constructed and sequenced at Shanghai Biozeron Biological Technology Co., Ltd. Briefly, for each sample, 1 μg of genomic DNA was sheared by Covaris S220 Focused-ultrasonicator (Woburn, MA, USA), and sequencing libraries were prepared with a fragment length of approximately 450 bp. All samples were sequenced using the Illumina NovaSeq 6000 platform at Shanghai Biozeron Biotechnology Co., Ltd., Shanghai, China.

Raw sequence reads underwent quality trimming using Trimmomatic v0.36 to remove adaptor contaminants and low-quality (quality below 20 and shorter than 50 bp) reads (Bolger et al., 2014). The taxonomy of clean reads for each sample was determined by Kraken2 using the customized kraken database. The abundances of taxonomy were estimated using Bracken (https://ccb.jhu.edu/software/bracken/) which can produce accurate species- and genus-level abundance even in multiple near-identical species. Clean sequence reads were assembled with MegaHit (v1.1.1-2-g02102e1). Assembled contigs were predicted using METAProdigal (v2.6.3), and a set of unique genes were generated using CD-HIT. Gene prediction was generated using MetaGeneMark software to identify coding regions in the genome. A non-redundant genome set (95% similarity threshold, 90% coverage threshold) was conducted using MMseq2 software. GO (Gene Ontology) annotation was performed using the *goatools* package. The unique gene set was first translated into protein sequences and then searched against the NCycle database (Tu et al., 2019) using DIAMOND (Buchfink et al., 2015) to identify the gene functions with the following filter parameters: evalue 0.00001, identity 90%. CAZymes were annotated using HMMER (v.3.2.1) to match the protein sequences to entries in the hidden Markov model (HMM) libraries of CAZyme (carbohydrate-active enzymes database) families downloaded from the CAZy (Lombard et al., 2014) database (http://www.cazy.org/, v12). KEGG (Kyoto Encyclopedia of Genes and Genomes) ortholog annotation was performed using KofamScan (https://www.genome.jp/tools/kofamkoala/) with the HMMSEARCH package (https://www.ebi.ac.uk/Tools/hmmer/search/hmmsearch).

### 2.5 Bioinformatic analyses

The composition of the rhizosphere bacterial community at the phylum level based on the OTUs (operational taxonomic units) data was generated using the *ggplot2* package in R (v 4.3.1). The α-diversity of the bacterial community was estimated using the non-parametric Shannon and Chao1 indices. A principal coordinate analysis (PCoA) based on Bray–Curtis distance metrics was performed with R (version 4.3.1) using the *vegan* package to explore differences in bacterial community compositions between clubroot-susceptible and clubroot-resistant cultivars of oilseed rape. The Bray–Curtis distance was generated based on OUT datasets at the genus level. Multivariate analysis of variance (MANOVA) was conducted based on Bray–Curtis distance metrics to further confirm the observed differences. The heatmap of the down- and upregulation of bacterial genera in the four cultivars of oilseed rape was calculated based on the relative abundance of each bacterial genera data in all the sequencing samples. Significance analysis of bacterial genera in the four cultivars of oilseed rape was generated using the STAMP software. Commonality analysis of variation in bacterial genera between the four cultivars was performed using an upset-venn diagram. The upset-venn diagram was completed using Wekomo Bioincloud (https://www.bioincloud.tech) (Gao et al., 2024).

**Figure 1:**
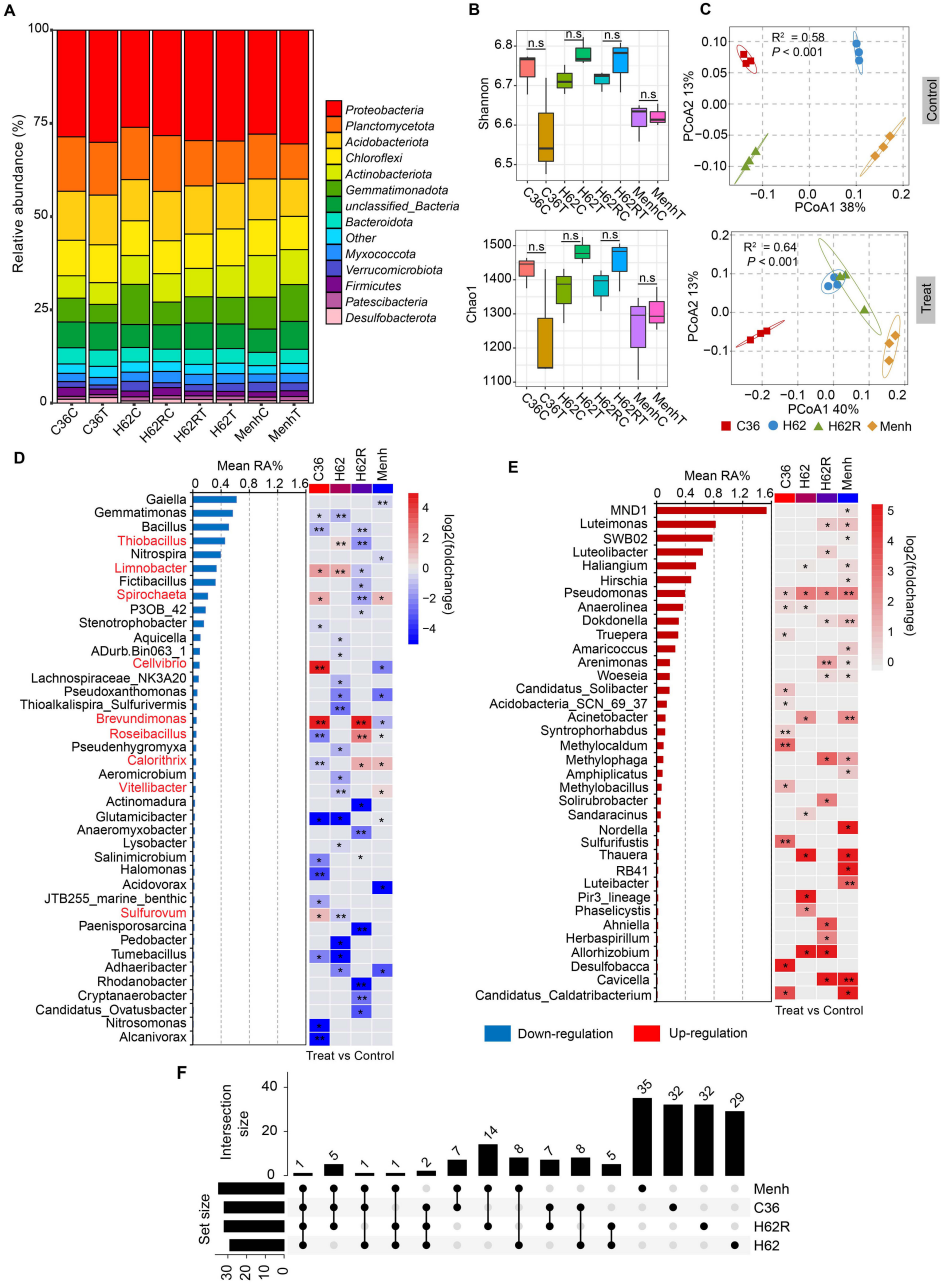
Comparison of rhizosphere bacterial diversity among the four of oilseed rape cultivars with or without *P. brassicae*. **(A)** Composition of rhizosphere bacterial community in the phylum level in all samples. C36C, H62C, H62RC, and MenhC means cultivars Chuanyou36, Huayouza62, Huayouza62R, and Menhir treated with water (Control). C36T, H62T, H62RT, and MenhT means four oilseed rape cultivars treated with resting spores of *P. brassicae*. **(B)** Rhizosphere bacterial α-diversity (Shannon and Chao1 indices) in all samples. “n.s.” means no significant difference between two samples (*P* > 0.05, *n* = 3, Student's *t*-test). **(C)** PCoA of rhizosphere bacterial community in C36, H62, H62R, and Menh of oilseed rape cultivars between the *P. brassicae*-treated and control groups. **(D)** Downregulation of bacterial genera in the four cultivars with *P. brassicae*-treated group compared with those of controls. “*, **” indicate significant differences among the samples (*P* < 0.05 and *P* < 0.01, *n* = 3, Student's *t*-test). **(E)** Upregulation of bacterial genera in the four cultivars with *P. brassicae* treatment compared with the controls. “*, **” indicate significant differences among the samples (*P* < 0.05 and *P* < 0.01, *n* = 3, Student's *t*-test). **(F)** Commonality analysis of variation in bacterial genera among the four cultivars based on the statistical results shown in **(E, F)**.

Based on the disease index of clubroot and commonality analysis of variation in bacterial genera between the four cultivars, C36 and H62 were classified as clubroot-susceptible types, and H62R and Menhir were classified as clubroot-resistant types (**Table 2**; **Figure 1F**). The averages of sequencing data from two cultivars representing the clubroot-susceptible or clubroot-resistant type data were used for the subsequent analysis. The bubble diagram was completed using Wekomo Bioincloud. Microbial co-occurrence networks were used to uncover the potential interactions between rhizosphere microbiomes for clubroot-susceptible and clubroot-resistant oilseed rape cultivars with or without *P. brassicae* treatment. For each treatment, we constructed one network to display the co-occurrence patterns of bacterial ASVs (amplicon sequence variants) in the rhizosphere with or without exposure to *P. brassicae* infection. Bacterial ASVs (with a relative abundance >0.1% for at least one sample) in the rhizosphere were selected for network construction. A pairwise Spearman correlation matrix was calculated with the “corr.test” function in the *psych* package in R (version 4.3.1). Robust correlations with Spearman’s correlation coefficients (*p*) > 0.6 or < -0.6 and *p* < 0.01 were used to construct networks. Network properties were performed in the *igraph* package in R (version 4.3.1). SIMPER (similarity percentages) analysis, which was completed based on the abundance data of bacterial ASVs using Wekomo Bioincloud, was used to identify the key bacterial genera that contribute significant differences in the rhizosphere bacterial co-occurrence network between susceptible and resistant cultivars. The mean relative abundance of bacterial genera was generated using the *ggplot2* package in R (version 4.3.1). Alpha and beta diversity of GO, KEGG, and CAZy pathways were performed using the *ggplot2* package in R (version 4.3.1). Significant difference analyses of the relative abundance of the KEGG pathway were completed using the STAMP software.

**Table 2:** Summary of clubroot disease indices in all the treatment groups in this study.

Sample	Treatment	Number of plants	Clubroot incidence (%)	Disease severity index
C36C	Control	63	**-**	**-**
H62C	Control	63	**-**	**-**
H62RC	Control	63	**-**	**-**
MenhC	Control	63	**-**	**-**
C36T	*P. brassicae*-treated	63	90.5 ± 7.8 a	74.1 ± 3.3 a
H62T	*P. brassicae*-treated	63	79.4 ± 6.0 a	61.4 ± 6.4 b
H62RT	*P. brassicae*-treated	63	39.7 ± 9.8 b	20.1 ± 6.5 c
MenhT	*P. brassicae*-treated	63	28.6 ± 11.7 b	11.1 ± 3.9 c

Different letters (a,b,c) represent significant differences among the treatments (Tukey test. P < 0.05, n = 3).

### 2.6 Data availability

The 16S rRNA amplicon data (SAMN40276299-SAMN40276322) and metagenome data (SAMN40350186-SAMN40350209) associated with this study have been deposited in the NCBI sequence read archive (SRA) under project accession PRJNA1084241. Source data have been provided in this article.

## 3 Results

### 3.1 Diversity of rhizosphere bacterial communities among the four oilseed rape cultivars infected with *P. brassicae*

The greenhouse experiment revealed that the four oilseed rape cultivars had varying susceptibility to *P. brassicae* infection. Data of CI and DSI showed that C36 and H62 cultivars were more susceptible than H62R and Menhir cultivars to *P. brassicae* infection (**Table 2**). We also tested rhizosphere bacterial community diversity in all treatments through 16S rRNA sequencing. A total of 794,568 16S rRNA gene reads were obtained from 16S rRNA sequencing data of all samples, with 2,277 bacterial zero-radius OTUs identified. The rhizosphere bacterial community in all samples was mainly dominated by phylum *Proteobacteria* (reads: 229,250), *Planctomycetota* (reads: 102,023), *Acidobacteriota* (reads: 96,102), *Chloroflexi* (reads: 68,968), *Actinobacteriota* (reads: 63,380), *Gemmatimonadota* (reads: 60,356), and *Bacteroidota* (reads: 31,054) (**Figure 1A**). The infection of *P. brassicae* did not significantly affect the rhizosphere bacterial α-diversity of oilseed rape at 40 dpi. Compared with the control group, the bacterial Shannon (6.58–6.78) and Chao1 (1,237–1,483) indices showed no significant difference in the four cultivars that were treated for *P. brassicae* (*P* > 0.05, **Figure 1B**). However, shifts in the rhizosphere bacterial communities were varied in the four cultivars under *P. brassicae* infection. PCoA analysis at the genus level indicated a significant difference in the structure of the rhizosphere bacterial community between C36 and Menhir cultivars in the control treatment, excluding H62 and H62R (**Figure 1C**). Consistently, MANOVA analysis confirmed that the cultivar type was the main driver of rhizosphere bacterial β-diversity under normal growth conditions (*R* ^2^ = 0.58, *P* < 0.001). However, compared with the control, the responses among the four cultivars differed when infected with *P. brassicae*, suggesting that the rhizosphere bacterial community changed in response to *P. brassicae* infection among the four cultivars (*R* ^2^ = 0.64, *P* < 0.001, **Figure 1C**).

Compared to the controls, the relative abundance of 76 (76/443, 17.2%) bacterial genera was significantly changed in the four cultivars when infected with *P. brassicae* (*P* < 0.05). A relative abundance of 40 bacterial genera was also significantly downregulated in the four cultivars when infected with *P. brassicae*, wherein nine bacterial genera underwent different changes in the four cultivars (**Figure 1D**). The rest of the 36 bacterial genera in the relative abundance level was significantly upregulated in the four cultivars (**Figure 1E**). The commonality analysis of variation in bacterial genera based on the upset-venn diagram showed that the four cultivars could be classified into two categories, C36 and H62 (top 2 intersection size: 8) and H62R and Menh (top 1 intersection size: 14) (**Figure 1F**)—for example, the relative abundance of *Gemmatimonas*, *Glutamicibacter*, and *Tumebacillus*, respectively, were significantly downregulated in the C36 and H62 cultivars. The relative abundance of *Limnobacter* was significantly upregulated in the C36 and H62 cultivars, while it was significantly downregulated in the H62R cultivar (**Figure 1D**). The relative abundance of *Luteimonas*, *Dokdonella*, *Arenimonas*, *Woeseia*, *Methylophaga*, and *Cavicella* were only significantly upregulated in the H62R and Menh cultivars (**Figure 1E**).

### 3.2 Differences in rhizosphere bacterial communities among different cultivars with varying susceptibility to infections by *P. brassicae*

**Figure 2:**
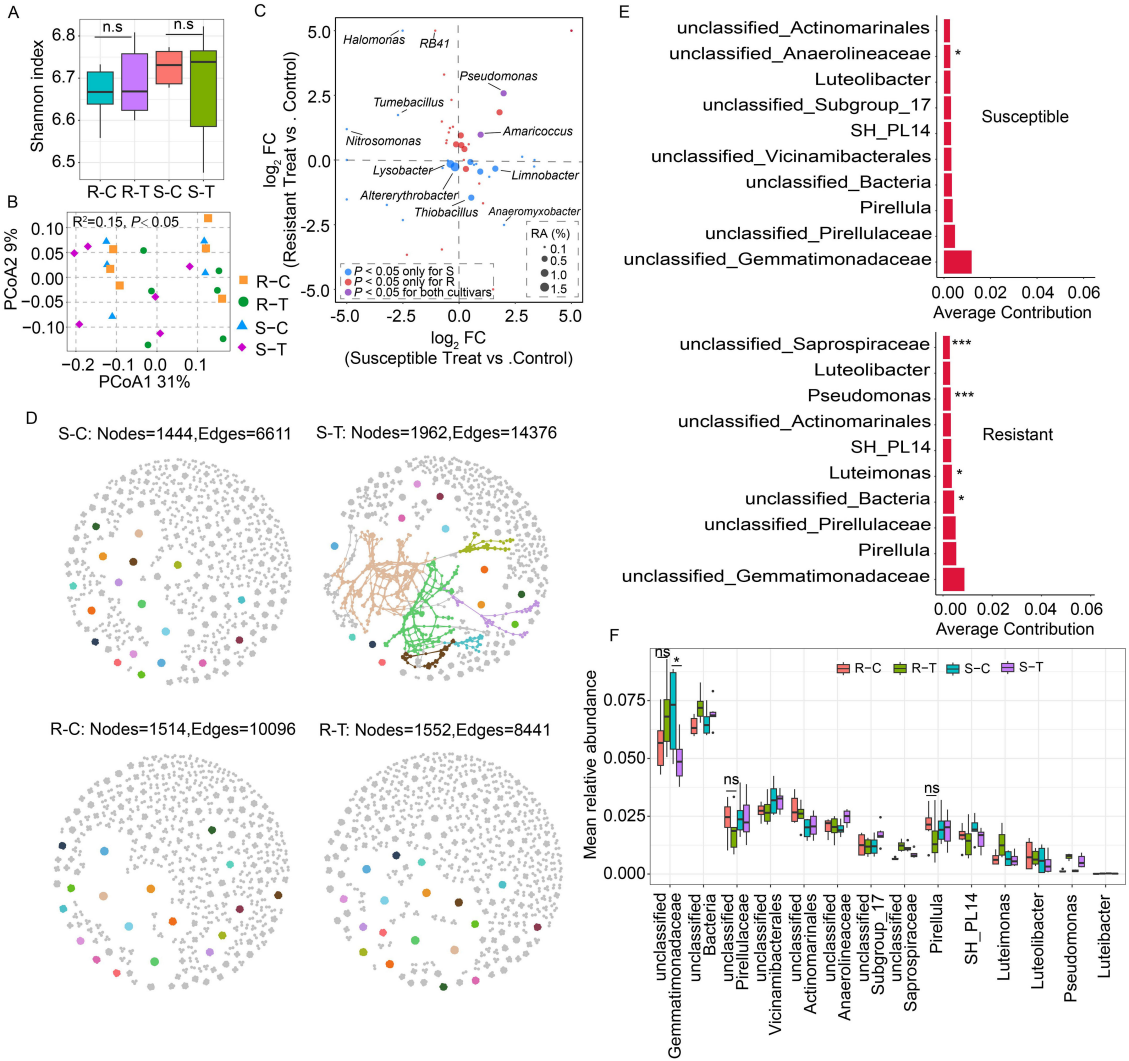
Comparison of rhizosphere bacterial community diversity between clubroot-susceptible and resistant oilseed rape cultivars. **(A)** Rhizosphere bacterial α-diversity (Shannon index) in clubroot susceptible cultivars treated with water (C36 and H62, S-C) and *P. brassicae* (S-T); clubroot-resistant cultivars treated with water (H62R and Menhir, R-C) and *P. brassicae* (R-T). “n.s.” means no significant difference between two samples (*P* > 0.05, *n* = 3, Student's *t*-test). **(B)** PCoA analysis of rhizosphere bacterial community structure between susceptible and resistant cultivars. **(C)** Comparison analysis of bacterial genera that underwent significant changes in the susceptible and resistant cultivars. FC means fold change in relative abundance level between the treated and control samples. RA means the relative abundance of bacterial genus in each sample. The blue dot means bacterial genera in relative abundance levels that only showed significant changes in susceptible cultivars. The red dot means bacterial genera in relative abundance levels that only showed significant changes in resistant cultivars. The purple dot indicates bacterial genera in relative abundance level that showed significant changes in both susceptible and resistant cultivars. **(D)** Rhizosphere bacterial co-occurrence networks between clubroot-susceptible and resistant oilseed rape cultivars treated with or without *P. brassicae*. **(E)** SIMPER analysis of the top ten bacterial genera that contribute to differences in the bacterial co-occurrence network in the susceptible and resistant cultivars between the control and *P. brassicae*-treated groups. *, *** indicate significant differences among the samples (*P*<0.05, *P*<0.005, n=6, Permutation test). **(F)** Mean relative abundance of the top 10 contributing bacterial genera in the susceptible and resistant cultivar samples. “ns” means no significant difference between two samples (*P* > 0.05, *n* = 6, Student's *t*-test). “*” indicate significant differences among the samples (P < 0.05, n = 6, Student’s t-test).

Two categories, clubroot-susceptible (C36 and H62) and clubroot-resistant cultivars (H62R and Menh), were classified based on DSI and commonality analysis of variation in rhizosphere bacterial genera for subsequent analysis (**Table 2**; **Figure 1F**). Compared with the control groups, the bacterial Shannon index showed no significant differences in both clubroot-susceptible and clubroot-resistant cultivars infected with *P. brassicae* (*P* > 0.05, **Figure 2A**). However, the bacterial β-diversity showed significant differences among the four groups (R-C, R-T, S-C, and S-T), indicating an obvious difference in bacterial community composition between them (*R* ^2^ = 0.15, *P* < 0.05, **Figure 2B**). Compared to the control group, a total of 49 bacterial genera with relative abundance levels were significantly changed in the clubroot-susceptible (*N* = 22) or clubroot-resistant (*N* = 24) cultivars under *P. brassicae* infestation (**Figure 2C**). Meanwhile, the relative abundance of *Pseudomonas* and *Amaricoccus* were significantly upregulated in both susceptible and resistant cultivars. Some bacterial genera, such as *Limnobacter*, *Thiobacillus*, and *Anaeromyxobacter*, were significantly upregulated in susceptible cultivars, while thy were downregulated in resistant cultivars. Furthermore, *Nitrosomonas*, *Tumebacillus*, and *Halomonas* were significantly downregulated in susceptible cultivars; however, these were upregulated in resistant cultivars.

Moreover, a rhizosphere bacterial co-occurrence network was generated to evaluate the interaction of rhizosphere bacterial communities of oilseed rape with or without *P. brassicae* infection. Compared to the control group (susceptible control, S-C), increased connectedness and robustness were exhibited in the rhizosphere bacterial community in susceptible cultivars infected with *P. brassicae* (susceptible treat, S-T) (**Figure 2D**). Consistently, the number of nodes and edges in the bacterial co-occurrence network in the S-T treatment was also higher than those in the S-C treatment group (S-C: nodes = 1,444, edges = 6,611, S-T: nodes = 1,962, edges = 14,376). On the contrary, similar changes were not observed in clubroot-resistant cultivars, while the connectedness and robustness of the bacterial co-occurrence network was slightly affected by *P. brassicae* infections (**Figure 2D**). The number of nodes and edges in the bacterial co-occurrence network showed a similar level between R-T and R-C treatments (R-C: nodes = 1,514, edges = 10,096; R-T: nodes = 1,552, edges = 8,441), while it was lower than those in the S-T treatment (**Figure 2D**). The above-mentioned results implied that *P. brassicae* infection had a significant impact on the interaction of the rhizosphere bacterial community in susceptible cultivars. The SIMPER analysis identified the top 10 bacterial genera responsible for differences in microbial co-occurrence network between susceptible and resistant cultivars (**Figure 2E**). Meanwhile, seven bacterial genera coexist in both susceptible and resistant cultivars, with the top three contributing bacterial genera being *unclassified_Gemmatimonadaceae*, *unclassified_Pirellulaceae*, and *Pirellula*. Compared to the controls, the relative abundance of *unclassified_Gemmatimonadaceae* was upregulated in resistant cultivars when subjected to *P. brassicae* infestation, while it was significantly downregulated in susceptible cultivars (*P* < 0.05) (**Figure 2F**). However, the relative abundance of *unclassified_Pirellulaceae* and *Pirellula*, respectively, were downregulated in resistant cultivars, while it was upregulated in susceptible cultivars (*P* > 0.05).

### 3.3 Functional diversity in the rhizosphere microbial community of clubroot-susceptible and clubroot-resistant oilseed rape cultivars exposed to *P. brassicae* infestation

**Figure 3:**
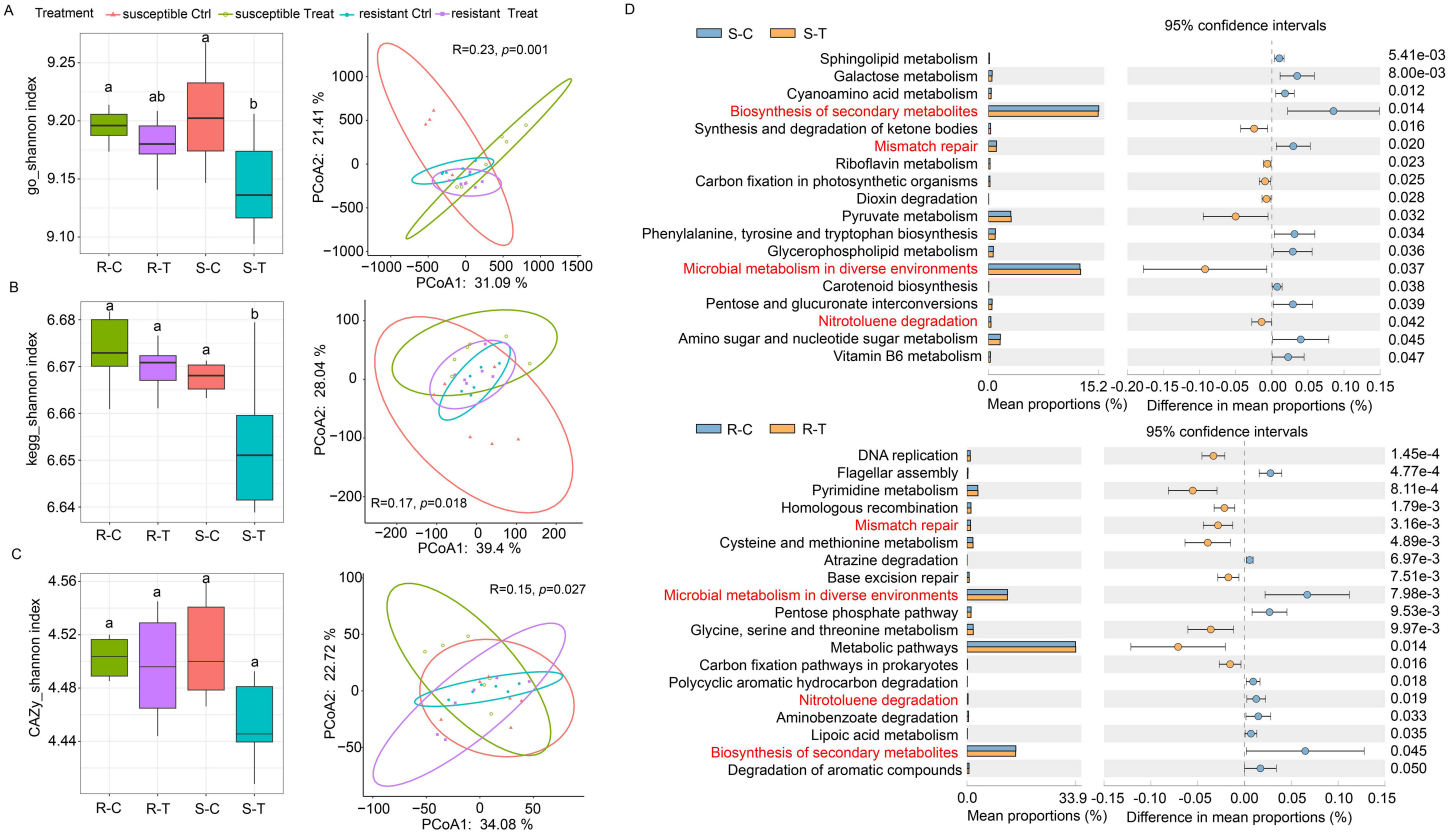
Functional diversity of rhizosphere microbiomes between clubroot-susceptible and resistant oilseed rape cultivars when exposed to *P. brassicae* infestation. **(A–C)** Functional diversity (Shannon index and PCoA) of rhizosphere microbiomes based on GO, KEGG, and CAZy databases between susceptible and resistant cultivars. Different letters represent significant differences among the treatments (*P* < 0.05, *n* = 6, Tukey test). **(D)** Differential KEGG pathways of rhizosphere microbiomes in the susceptible and resistant cultivars between the control and *P. brassicae*-treated groups.

Metagenomic analysis was used to investigate functional differences in the rhizosphere microbial community between clubroot-susceptible and clubroot-resistant cultivars when exposed to *P. brassicae* infection. An analysis of functional diversity annotated based on GO and KEGG databases showed that there was a significant difference between susceptible and resistant cultivars with or without *P. brassicae* infestation. Compared to the controls, rhizosphere microbial functional α- and β-diversity based on GO and KEGG databases showed significant changes in susceptible cultivars exposed to *P. brassicae* infection, while there was no significant change in resistant cultivars (**Figures 3A, B**). In contrast to the two types previously mentioned, rhizosphere microbial functional α- and β-diversity based on CAZy database showed no significant changes in both of the two cultivars exposed to *P. brassicae* infection compared with the controls (**Figure 3C**).

An analysis of KEGG pathways showed that the relative abundance of multiple pathways was significantly changed in susceptible and resistant cultivars under *P. brassicae* infection compared with the controls. In susceptible cultivars, the relative abundance of 11 pathways were significantly downregulated when exposed to *P. brassicae* infection compared with the controls, while the other seven pathways were significantly upregulated (**Figure 3D**). In the resistant cultivars, the relative abundance of 10 pathways was significantly downregulated when exposed to *P. brassicae* infection compared with the controls, while the other nine pathways were significantly upregulated (**Figure 3D**). Among them, most of the pathways belonged to microbial metabolism, and three pathways (biosynthesis of secondary metabolites, microbial metabolism in diverse environments, and nitrotoluene degradation) showed significant changes in both susceptible and resistant cultivars. However, microbial metabolism in diverse environments and nitrotoluene degradation pathways showed opposite changes between susceptible and resistant cultivars. Both of the two pathways were significantly upregulated in the susceptible cultivars under *P. brassicae* infection compared with the controls, while they were significantly downregulated in resistant cultivars.

**Figure 4:**
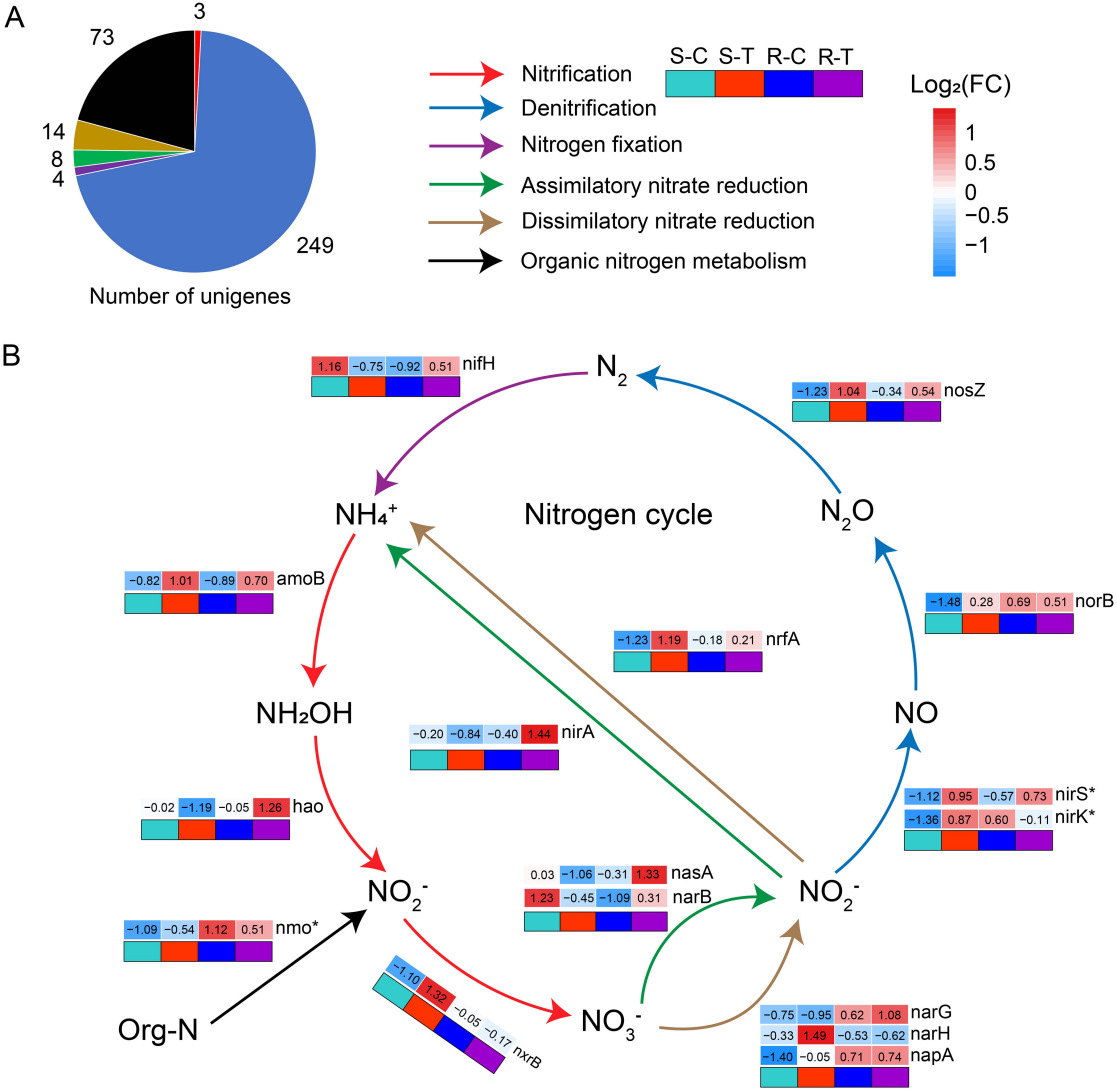
Metagenomic analysis of nitrogen cycle pathways of rhizosphere microbiomes in susceptible and resistant cultivars. **(A)** Number of unigenes annotated to genes related to the nitrogen cycle pathway. The color of the cake is consistent with that shown in **(B)**, indicating the type of nitrogen cycle pathway. **(B)** The red arrow indicates the nitrification pathway (*amoB*, *hao*, and *nxrB*) of the nitrogen cycle. The blue arrow indicates the denitrification pathway (*nirS*, *nirK*, *norB*, and *nosZ*) of the nitrogen cycle. The purple arrow indicates the nitrogen fixation pathway (*nifH*) of the nitrogen cycle. The green arrow represents the assimilatory nitrate reduction pathway (*nasA*, *narB*, and *nirA*) of the nitrogen cycle. The brown arrow indicates the dissimilatory nitrate reduction pathway (*narG*, *narH*, *napA*, and *nrfA*) of the nitrogen cycle. The black arrow represents the organic nitrogen metabolism pathway (*nmo*) of N cycle. “*” indicates significant differences among the samples (*P* < 0.05, *n* = 6, Student's *t*-test).

Subsequently, we focused on the variation of genes and pathways in the nitrogen (N) cycle between susceptible and resistant cultivars with or without *P. brassicae* infection. A total of 351 unigene protein sequences (identity ≥90%) were matched to 23 genes involved in the N cycle pathway, including nitrification (*amoB*, *hao*, and *nxrB*; number of unigenes = 3), denitrification (*nirS*, *nirK*, *norB*, and *nosZ*; number of unigenes = 249), nitrogen fixation (*nirH*; number of unigenes = 4), assimilatory nitrate reduction (*nasA*, *narB*, and *nirA*; number of unigenes = 8), dissimilatory nitrate reduction (*narG*, *narH*, *napA*, and *nrfA*; number of unigenes = 14), and organic nitrogen metabolism (*nmo*, gdh_K00261, gdh_K00262, gdh_K15371, *glsA*, *glnA*, *ureA*, and *ureC*; number of unigenes = 73) (**Figure 4A**). Among them, 16 genes were further analyzed. Compared to the controls, multiple genes showed different changes between susceptible and resistant cultivars when exposed to *P. brassicae* infection. In susceptible cultivars, we observed a higher abundance of genes associated to nitrification (*amoB* and *nxrB*), dissimilatory nitrate reduction (*narH* and *nrfA*), and denitrification (*nirS*, *nirK*, *norB*, and *nosZ*) when exposed to *P. brassicae* infection compared with the controls (**Figure 4B**). A variation in the *nxrB* gene abundance in the nitrification pathway (NO_2_^-^→NO_3_^-^) indicated an accelerated process in NO_3_^-^ synthesis, which was consistent with the results above (**Figure 2**). However, in the resistant cultivars, a number of genes associated with assimilatory nitrate reduction (*nasA*, *narB*, and *nirA*), denitrification (*nirS* and *nosZ*), nitrogen fixation (*nirH*), and nitrification (*amoB* and *hao*) were upregulated when infected with *P. brassicae* compared with the controls, while the other genes associated with denitrification (*nirK*) and organic nitrogen metabolism (*nmo*) were downregulated. Additionally, a variation in the abundance of *nasA*, *narB*, and *nirA* genes in the assimilatory nitrate reduction pathway indicated an accelerated process in NO_3_^-^ assimilation.

## 4 Discussion

Alterations in the rhizosphere microbiome always occur in plants after being infected by soil-borne pathogens; this phenomenon has been reported in several crop/pathogen disease systems, including clubroot (Kwak et al., 2018; Hu et al., 2020; Ni et al., 2022). However, studies on the differences in response of rhizosphere bacterial community to *P. brassicas* infection between clubroot-susceptible and clubroot-resistant cultivars are limited. Herein we investigated the variation in rhizosphere bacterial community in oilseed rape cultivars with different susceptibility to *P. brassicae* infection. Our results revealed a distinct shift in rhizosphere bacterial communities in the four cultivars when infected with *P. brassicae* compared with the control, and this also varied among the cultivars (**Figure 1**). The results of this study indicated that the response of rhizosphere bacterial community to *P. brassicae* infection was different among the four cultivars, and the cultivar type was the main driving factor that led to variations in the rhizosphere bacterial community (**Figure 1**). Our results are consistent with those of previous studies, indicating that the host genotype has a significant impact on shaping the plant rhizosphere microbial community, which has also been extensively confirmed in other crops (Zhang et al., 2019)—for example, the Indica variety of rice showed a higher nitrogen use efficiency than that of the Japonica variety through recruiting more microbial taxa with nitrogen metabolism functions, which is determined by a nitrate transporter and sensor named *NRT1.1B*. Moreover, this distinct shift in rhizosphere bacterial community is a result of the interaction between the host plant and *P. brassicae*. Previous studies have also proven that a distinct transcriptome landscape existed in the roots of clubroot-susceptible and clubroot-resistant Chinese cabbage lines after *P. brassicae* infection, indicating an obvious difference in clubroot resistance mechanisms and root exudations (Jia et al., 2017). A variation in the root transcription landscape could also lead to alterations in root metabolites, which, in turn, may affect the rhizosphere bacterial community (Pedras et al., 2008; Li et al., 2022). Moreover, shaping plant rhizosphere microbiomes can be achieved through the secretion of root exudates (Hu et al., 2018). In cereal crops such as wheat and maize, plant root would release benzoxazinoids to alter root-associated fungal and bacterial communities under a pathogen’s infestation.

Although four cultivars showed significant differences in their rhizosphere bacterial community when infected with *P. brassicae*, clubroot occurrence performance and commonality analysis of variation in bacterial genera also confirmed that cultivars C36 and H62, H62R, and Menhir, could be classified into clubroot-susceptible and clubroot-resistant types (**Table 2**; **Figure 1**). A comparative analysis further revealed differences in rhizosphere bacterial communities between clubroot-susceptible and clubroot-resistant cultivars in response to *P. brassicae* infection (**Figure 2**). Compared to the controls, some bacterial genera, such as *Limnobacter*, *Thiobacillus*, *Anaeromyxobacter Nitrosomonas*, *Tumebacillus*, and *Halomonas*, showed significant changes in relative abundance level between susceptible and resistant cultivars when exposed to *P. brassicae* infection. Meanwhile, *Limnobacter*, *Thiobacillus*, and *Anaeromyxobacter* were reported to be associated with BNF (biological N_2_ fixation) and were significantly upregulated in susceptible cultivars infected with *P. brassicae* and were downregulated in resistant cultivars. Additionally, *Thiobacillus* and *Anaeromyxobacter* were reported to be associated with arsenite oxidation-dependent biological nitrogen fixation, and *Limnobacter* was reported to be associated with nitrification-anammox (PN/A) processes (Li et al., 2023; Wang P. et al., 2024). Our results suggested that variations in the relative abundance of those three bacterial genera may lead to an accumulation of NO_3_^-^ and SO_4_^2-^ in the soil and a reduction in pH value, which may be conducive to *P. brassicae* infection by reducing the soil’s pH value (Wang et al., 2023). Moreover, *Nitrosomonas*, *Tumebacillus*, and *Halomonas* were significantly downregulated in susceptible cultivars but were upregulated in resistant cultivars. *Nitrosomonas*, *Tumebacillus*, and *Halomonas* were reported to be associated with nitrosation and aerobic denitrification; variations in the relative abundance of those bacterial genera have also been shown to lead to an accumulation of NO_3_^-^ and SO_4_^2-^ in soil samples from susceptible cultivars (Arp et al., 2002; Zhang et al., 2014; González-Domenech et al., 2010).

A variation in bacterial taxa is also reflected in microbial interactions. The bacterial co-occurrence network also showed a distinct interaction intensity within rhizosphere bacterial communities in susceptible cultivars infected with *P. brassicae* compared with the controls. Meanwhile, increased connectedness and robustness of the bacterial co-occurrence network were observed in susceptible cultivars exposed to *P. brassicae* infestation compared with the controls, while there were slight changes in resistant cultivars. The SIMPER analysis revealed that *unclassified_Gemmatimonadaceae*, *unclassified_Pirellulaceae*, and *Pirellula* were the three most common bacterial genera that contributed to the differences in bacterial co-occurrence network between the *P. brassicae*-treated and control samples in susceptible and resistant cultivars (**Figure 2E**). Among them, the relative abundance of *unclassified_Gemmatimonadaceae* was upregulated in resistant cultivars exposed to *P. brassicae*, while it was significantly downregulated in susceptible cultivars (**Figure 2F**). *Gemmatimonadaceae* was reported to be associated with plant root metabolites, which had a negative correlation with organic acid and a positive correlation with ketone content (Wang M. et al., 2024). Flavonoids, a type of ketone, have been reported to be associated with clubroot disease resistance in *Arabidopsis thaliana* (Päsold et al., 2010). The relative abundance of *unclassified_Pirellulaceae* and *Pirellula* was downregulated in the resistant cultivars when exposed to *P. brassicae*, while it showed slight changes in susceptible cultivars. *Pirellula* belonged to *Planctomycetes* and has been shown to be associated with anaerobic ammonia oxidation (Huang et al., 2014). In this study, a decrease in the relative abundance of *Pirellula* in resistant cultivars may also lead to the degradation of NO_3_^-^ in the soil.

Variations in bacterial taxa and interaction within rhizosphere bacterial communities may also be reflected in microbial metabolisms. Functional diversity analysis based on metagenomic sequencing data further confirmed that changes in bacterial communities resulted in alterations in microbial metabolism. Our results showed that multiple pathways associated with microbial metabolism were significantly different in susceptible and resistant cultivars when exposed to *P. brassicae* infection compared with the controls (**Figure 3**). Among them, pathways associated with the nitrogen cycle exhibited distinct differences between susceptible and resistant cultivars (**Figure 3D**). Our results also demonstrated that the synthesis (nitrification) and assimilation (assimilatory nitrate reduction) processes of NO_3_^-^ content were promoted in susceptible and resistant cultivars, respectively. Meanwhile, the abundance of *nxrB* gene related to nitrification (NO_2_^-″^NO_3_^-^) was upregulated in susceptible cultivars exposed to *P. brassicae* infection compared with the control, while it only changed slightly in resistant cultivars. Moreover, the expression of *nasA*, *narB*, and *nirA* genes, which are related to assimilatory nitrate reduction, was upregulated in resistant cultivars when exposed to *P. brassicae* infections compared with the controls, while it was downregulated in susceptible cultivars (**Figure 4**). In this study, the differences in nitrification and assimilatory nitrate reduction pathways between susceptible and resistant cultivars were due to rhizosphere microbiomes in response to *B. napus*/*P. brassicae* interaction. Our results suggested that NO_3_^-^ may be one of the critical factors that affect *B. napus*/*P. brassicae* interaction and could reduce the incidence of clubroot.

Nitrogen, which is widely considered as a central element in soil ecosystems, has a huge impact on plant/pathogen interactions (Cui et al., 2014). Meanwhile, nitrogen supply could enhance the development of biotrophic pathogens, while the opposite effect is observed for necrotrophic pathogens (Solomon et al., 2003; Mur et al., 2016). The different forms of nitrogen supply (ammonium NH_4_^+^ or nitrate NO_3_^-^) can have various effects on the occurrence of plant disease due to differences in assimilation and metabolism pathways (Mur et al., 2016). NO_3_^-^ feeding can strengthen host hypersensitive response (HR)-mediated resistance through enhancing the production of polyamines, while NH_4_^+^ nutrition can attenuate host defense. Regarding clubroot, although some studies suggest that the occurrence of clubroot is reduced with the application of high-nitrogen fertilizers, this may be attributed to the fact that oilseed rape requires a relatively large amount of nitrogen fertilizer during its entire growth period for growth and disease resistance (Gossen et al., 2014; Rathke et al., 2005). However, recent studies have shown that a high nitrogen supply could promote the occurrence of clubroot in susceptible *B. napus* cultivars by regulating the transcriptomic profile of *P. brassicae*, including pathogenicity-related genes (NUDIX and NEP-proteins) and genes associated to obligate biotrophic functions (glutamine synthetase, associated with nitrogen metabolism), whereas the effect differs in resistant cultivars (Gazengel et al., 2021). The above-mentioned results suggest that nitrogen supply may be beneficial for *P. brassicae* infection. Germination of *P. brassicae*’s resting spores is crucial for the occurrence of clubroot disease. Recent studies have proven that a diverse bacterial community, rather than root exudates, is necessary to stimulate the germination of the resting spores of *P. brassicae* (Wang et al., 2023). Meanwhile, the relative abundance of *Sphingobacteriia*, *Flavobacteriia*, and *Bacteroidetes* was significantly enriched in the “high”-germination-rate group, while *Proteobacteria* dominated in the “low”-germination-rate group. Moreover, the addition of NO_3_^-^, not NH_4_^+^, was conducive for the induction of the microbial community, leading to the germination of resting spores. The NO_3_^-^ supply may be utilized as nutrients by certain microorganisms and could enhance nitrogen cycle pathways within microbial communities. The results of this study are consistent with the conclusions of previous studies. The NO_3_^-^ synthesis pathways in the rhizosphere microbiomes were promoted in susceptible cultivars when exposed to *P. brassicae* infection compared with the controls, while the NO_3_^-^ assimilation pathways in the rhizosphere microbiomes were promoted in resistant cultivars. Furthermore, in this study, variations in the NO_3_^-^ assimilation and synthesis pathways in rhizosphere microbiomes were a result of the occurrence of clubroot, suggesting that changes in rhizosphere microbial community were directed by the *B. napus*/*P. brassicae* interaction. We considered that this rhizosphere microbial ecology environment associated with NO_3_^-^ accumulation in the susceptible cultivar may be conductive to the further development of clubroot. In the clubroot-susceptible cultivar, once the host’s own defense system was breached, *P. brassicae* may control the rhizosphere microbial community to facilitate further infection by regulating the host’s metabolism. However, in clubroot-resistant varieties, the rhizosphere microbial community may be continuously manipulated by the host to jointly resist the *P. brassicae* infection. The differences between susceptible and resistant cultivars may be determined by their distinct resistance mechanisms. Multiple studies have also shown that *Brassica* crop roots showed different changes in genes, transcription, metabolomics, and proteome perspectives after *P. brassicae* infection, while the situation varied between susceptible and resistant cultivars due to the CR gene (Chen et al., 2015; Zhang et al., 2016; Pedras et al., 2008; Cao et al., 2008; Li et al., 2022). In this study, although we revealed the differences in NO_3_^-^ assimilation and synthesis pathways in the rhizosphere microbiomes between susceptible and resistant cultivars after infection with *P. brassicae*, it is still unclear that soil NO_3_^-^ participated in the interaction between hosts, *P. brassicae*, and rhizosphere microbiomes. In this study, the results of microbial diversity were conducted based on relative abundance indicators, and we only focused on limited microbial taxa that were differentially distributed among different samples. Hence, we might ignore those microbial taxa that showed significant changes in absolute abundance level across different samples while having similar relative abundance levels, and these microbes might be crucial for regulating microbial ecological functions. Moreover, all the conclusions obtained in this study were completed in the greenhouse condition, so several environmental impact factors, such as soil property, fertilizer regime, and cultivation pattern, were overlooked compared to field experiments. Our further study will validate the effect of NO_3_^-^ pathway of microbial community on clubroot occurrence and clarify the relationship between soil NO_3_^-^ content and microbial community function. We will also investigate the succession pattern of soil nitrogen cycling during the occurrence of the clubroot disease process, which is conducive to clarify the occurrence mechanism of clubroot disease and lay a theoretical foundation for clubroot disease control by using soil microorganisms.

## Data Availability

The datasets presented in this study can be found in online repositories. The names of the repository/repositories and accession number(s) can be found below: https://www.ncbi.nlm.nih.gov/, PRJNA1084241.
